# Robotic Assisted Laparoscopic Prostatectomy in Men with Proctocolectomy and Restorative Ileal Pouch-Anal Anastomosis

**DOI:** 10.1155/2014/538382

**Published:** 2014-02-05

**Authors:** Michael Leapman, Young Suk Kwon, Shemille A. Collingwood, Edward Chin, Maria Katsigeorgis, Adele R. Hobbs, David B. Samadi

**Affiliations:** ^1^Department of Urology, Icahn School of Medicine at Mount Sinai, 1425 Madison Avenue, 6th Floor, New York, NY 10029, USA; ^2^Mercer University School of Medicine, 1400 Coleman Avenue, Macon, GA 31207, USA; ^3^Department of General Surgery, Icahn School of Medicine at Mount Sinai, 5 East 98th Street, 14th Floor, P.O. Box 1259, New York, NY 10029, USA; ^4^Department of Urology, North Shore LIJ, Lenox Hill Hospital, 485 Madison Avenue; 21st Floor, New York, NY 10022, USA; ^5^Helen Diller Family Comprehensive Cancer Center, University of California San Francisco, 1600 Divisadero Street, Room B-708, San Francisco, CA 94115, USA

## Abstract

We conducted a retrospective chart review of robotic prostatectomies done by a single surgeon between 2003 and 2012. During that time period, we identified two patients within the year 2012, with ileal pouch-anal anastomosis (IPPA) who also underwent robotic prostatectomies. The demographics and postoperative characteristics of the two patients were assessed. In both patients, prostatectomy, bilateral nerve sparing, and pelvic lymphadenectomy were successfully performed and the integrity of ileal pouch was maintained. There was a mean surgical time of 144.5 minutes, and an average estimated blood loss was 125 mL. Both patients were discharged on the second day postoperatively. In both patients there was a Gleason upgrade to 3 + 4, with negative margins, and preservation of fecal and urinary continence by their six-month followup. Owing to surgical modifications, these two surgeries represent the first successful robotic prostatectomies in patients with a J-pouch.

## 1. Introduction

Proctocolectomy with restorative ileal pouch-anal anastomosis (PC-IPAA) is commonly utilized for the treatment of familial adenomatous polyposis and medication-refractory ulcerative colitis, where long-term followup has demonstrated favorable functional and quality of life outcomes [1, 2]. Screening and treatment recommendations for men with prior history of PC-IPAA remain to be definitively assessed; however, radiation therapy is typically avoided due to concerns regarding injury to the pouch. Radical prostatectomy for localized prostate cancer presents unique challenges and has conventionally been performed using an open retropubic approach [3]. We present our surgical technique, outcomes, and feasibility assessment following robotic assisted laparoscopic prostatectomy (RALP).

## 2. Case Report

Two patients with history of PC-IPAA for ulcerative colitis (UC) underwent RALP for clinically localized prostate cancer detected by PSA surveillance. The mean preoperative PSA was 9.1 ng/mL, and the mean age was 61 years. Both patients underwent transperineal prostate biopsies that demonstrated high volume Gleason score 3 + 3 = 6 prostate adenocarcinoma. The operative approach included modified trocars placement under direct visualization, with additional assistant port used for extensive lysis of adhesions. Significant adhesions were encountered posteriorly with near encasement of seminal vesicles. Prostatectomy, bilateral nerve sparing, and pelvic lymphadenectomy were successfully performed in both patients. The integrity of ileal pouch was confirmed with gentle insufflation of air under vision. Mean surgical time was 144.5 minutes, and the average estimated blood loss was 125 mL. Mean time to flatus was two days, and both patients were discharged on postoperative day two. In both patients Gleason score was upgraded to 3 + 4, negative margins were obtained, and fecal and urinary continence were preserved by six-month followup.

## 3. Conclusion

RALP in patients with ileal pouch-anal anastomosis (IPPA) is feasible, oncologically efficacious, yet technically challenging. Obliteration of Denonvilliers' fascia should be anticipated, and care is taken to minimize dissection in proximity of ileal pouch. This represents the first report of successful RALP in these patients. Minimally invasive prostatectomy may gain increasing utilization by experienced robotic surgeons for patients with inflammatory bowel disease or familial adenomatous with proctocolectomy and restorative ileoanal pouch.

## 4. Discussion

Ileal pouch-anal anastomosis (IPPA) remains the surgical procedure of choice for chronic ulcerative colitis and familial adenomatous polyposis on account of several factors. This procedure allows for gastrointestinal continuity and preservation of the anal sphincter, the elimination of the need for permanent stoma, and improvement in quality of life. Presently, there are as many as 1.4 million people in the USA suffering from some type of inflammatory bowel disease (IBD). Unsurprisingly, there is an increasing number of patients who are affected by both prostate cancer (PCA) and IBD. Patients with IPAA-PCA have historically been considered poor candidates for radical prostatectomy owing to loss of anatomic tissue, planes, denervation of pelvic musculature, and pelvic adhesions. Surgery has traditionally been performed retropubically, through an “open” approach. Similarly, external beam radiotherapy, another treatment option, is associated with significant risk to overlying J-pouch and has been avoided. Brachytherapy may be a viable treatment option, but negative side effects should not be overlooked [4].

With a growing population of men treated with proctocolectomy and J-pouch, incidence of prostate cancer in otherwise surgically suitable men is expanding. Although this is the first case series of successful RALP in patients with J-pouch, our initial experience suggests that minimally invasive robotic prostatectomy may be offered to men with both clinically significant prostate cancer and J-pouch. It represents an advanced level procedure that requires considerable comfort and experience, but we anticipate that it will gain increasing utilization by experienced robotic surgeons for patients with inflammatory bowel disease or familial adenomatous with proctocolectomy and restorative ileoanal pouch.

Given the complexity of the case, both a skilled robotic surgeon and a skilled general surgeon are necessary to complete this case successfully. Both surgeons should be experienced in their fields in order to appropriately adjust their surgical techniques to accommodate a wide variety of possible anatomic variants while still providing an effective treatment. While ileoanal anastomosis may make a RALP technically difficult, we have shown that in the hands of accomplished surgeons it can be done with positive outcomes.

## References

[1] McGuire BB, Brannigan AE, O’Connell PR (2007). Ileal pouch-anal anastomosis. *British Journal of Surgery*.

[2] Michelassi F, Lee J, Rubin M (2003). Long-term functional results after ileal pouch anal restorative proctocolectomy for ulcerative colitis: a prospective observational study. *Annals of Surgery*.

[3] Umbreit EC, Dozois EJ, Crispen PL, Tollefson MK, Karnes RJ, Blute ML (2010). Radical prostatectomy for prostate cancer after ileal pouch-anal anastomosis offers oncologic control and sustains quality of life. *Journal of the American College of Surgeons*.

[4] Williamson R, Smaldone MC, Gibbons EP, Smith RP, Beriwal S, Benoit RM (2009). Prostate brachytherapy after ileal pouch-anal anastomosis reconstruction. *Urology*.

